# Habitat isolation reduces intra- and interspecific biodiversity and stability

**DOI:** 10.1098/rsos.211309

**Published:** 2022-02-16

**Authors:** Christopher F. Steiner, Mitra Asgari

**Affiliations:** Department of Biological Sciences, Wayne State University, 5047 Gullen Mall, Detroit, MI 48202, USA

**Keywords:** dispersal, diversity, eco-evolutionary dynamics, fragmentation, metacommunity dynamics, stability

## Abstract

Fragmentation is predicted to reduce biodiversity and stability by increasing habitat isolation and impeding dispersal among patches. These effects may manifest at both the interspecific and intraspecific levels, yet few studies have simultaneously explored dispersal effects across levels of organization. We used field mesocosm experiments to examine how habitat isolation (in the form of dispersal rate) alters inter- and intraspecific stability and diversity in local zooplankton communities. We observed effects of increasing dispersal rate at both the intra- and interspecific levels. Increasing dispersal increased local species diversity and reduced mean temporal variability of populations. At the intraspecific level, *Daphnia pulex* clonal diversity was enhanced by dispersal and mean temporal variability of clone abundances through time was reduced.

## 1. Introduction

Of the factors known to threaten biodiversity, habitat loss and fragmentation have had the largest impact across the broadest range of biomes in the last 50 to 100 years [1–5]. Fragmentation may alter biotic systems by increasing habitat isolation and impairing the movement of organisms among remnant patches. Resultant reductions in dispersal capacity can, in theory, reduce dynamic stability and diversity measured at both intra- and interspecific levels. Yet, controversy remains regarding the importance of habitat isolation in nature when compared to other effects of fragmentation such as reductions in local habitat size [6]. Furthermore, experimental studies have rarely considered concurrent effects of spatial isolation on both interspecific responses and intraspecific, eco-evolutionary responses. Genetic diversity and its maintenance through time are recognized as potentially vital factors influencing the persistence of populations [7]. Thus, comprehending human impacts on the integrity and long-term persistence of biological systems may depend on understanding simultaneous impacts at multiple levels of organization.

Increasing habitat isolation can impact local populations and communities by reducing dispersal among local patches [6,8]. In their seminal model of metacommunity dynamics, Wilson & MacArthur [9] equated increasing spatial isolation of insular communities with reduced immigration rates and generated the well-known prediction that local species diversity declines with increasing isolation. Models since have shown that decreased rates of movement among patches may have significant negative impacts on both intra- and interspecific stability and biodiversity. A common model prediction is that dispersal can reduce the extinction probability of local populations by increasing population abundances and reducing their variability through time [10–13]. Dispersal may also enhance stability and persistence of local populations by augmenting population growth rates during transitory periods of positive exponential growth [12,14]. At the community level, dispersal can also enhance local population persistence and species diversity through alterations of competitive outcomes, reducing competitive exclusion and sustaining sub-dominant competitors within patches [15,16]. Hence, dispersal can simultaneously enhance temporal stability of populations and local species diversity.

In addition to its ecological impacts, dispersal and gene flow may influence the evolutionary dynamics of populations—enhancing adaptive capacity, mean fitness and long-term persistence of populations under certain circumstances [17–23]. Low to intermediate levels of gene flow can also increase and stably maintain genetic diversity within populations through time [24]. Moreover, spatial isolation and reduced gene flow can increase the effects of genetic drift, eroding local genetic diversity [22,25,26]. Thus, much like ecological effects, dispersal can promote genetic diversity and the stability of gene frequencies within patches through time. Despite this, few studies have simultaneously explored its impacts at both intra- and interspecific levels. This likely stems from the historical perspective that evolutionary dynamics occur at time scales that are much longer than ecological dynamics. For organisms with short generation times such as zooplankton, rapid evolutionary responses are possible and readily evident in natural and experimental settings [27–32]. Consequently, concurrent effects of reduced dispersal on stability and biodiversity may be apparent both among and within species' populations at ecological time scales.

Here, we present the results of a field mesocosm experiment in which we tested the effects of habitat isolation on local biodiversity and temporal stability in freshwater zooplankton assemblages. We focused on one key component of habitat isolation—its effects on emigration/immigration rates—and directly manipulated reciprocal dispersal rates among patches while maintaining constant patch size. Treatment effects on genetic composition were measured for a single species, *Daphnia pulex*, a common and often numerically dominant taxon in natural ponds in the study region [33]. Our results supported general predictions that decreasing isolation and increasing dispersal among patches can increase local diversity and stability both among species and within *D. pulex* populations.

## 2. Methods

### 2.1. Fieldwork

The experiment was conducted at the W. K. Kellogg Biological Station experimental pond facility (Hickory Corners, MI, USA). This research did not require ethical approval or permits prior to it being conducted. Mesocosms consisted of polyethylene tanks filled with 300 l of untreated well water and covered with 1 mm screening. We added phosphorus and nitrogen as K_2_HPO_4_ and NaNO_3_ to concentrations of 150 µg P l^−1^ and 2250 µg N l^−1^, comparable to median levels found in ponds in the region [33]. All tanks were inoculated with phytoplankton collected from eight ponds near the field station. To collect phytoplankton, two litres of water were collected at mid-depth from the centre of each pond and filtered through a 35 µm mesh to remove macrozooplankton. Water samples were then pooled, mixed and 250 ml added to each tank. The zooplankton assemblage consisted of six species that are common and numerically dominant cladocera in ponds in the region [33]: *Bosmina longirostris*, *Ceriodaphnia reticulata*, *Chydorus sphaericus*, *Daphnia pulex*, *Diaphanosoma brachyurum* and *Scapholeberis mucronata*. Hereafter, we refer to these species by genus. All taxa were collected from local ponds and maintained in the laboratory under common garden conditions for more than four generations prior to the start of the experiment. We used 10 *D. pulex* clones that were collected from different ponds in the region and maintained in the laboratory. Clones were identifiable as unique multi-locus genotypes using four microsatellite markers. The remaining five zooplankton taxa were cultured as isogenic lines and likely exhibited little or no genetic variation at the initiation of the experiment. One week following phytoplankton addition, 20 individuals of each *Daphnia* clone and each non-*Daphnia* zooplankton taxon were added to each mesocosm. We refer to this as day 0 of the experiment.

The experimental design consisted of four levels of decreasing habitat isolation in the form of dispersal among tanks (0%, 3.33%, 10% and 20% by volume, twice weekly). Local communities that experienced no dispersal (full habitat isolation) were single tanks. Non-zero dispersal treatments occurred within metacommunities each composed of three tanks. All metacommunities and dispersal absent tanks were replicated four times for a total of 40 tanks. Placements of replicate 0% dispersal tanks and replicate metacommunities were completely randomized in the field. Dispersal treatments consisted of density-independent dispersal events performed every 2–3 days by gently mixing each tank in a metacommunity and removing the appropriate percentage of volume using a 10 l bucket. Zooplankton were filtered out using a 60 µm sieve and the water was returned to its source tank. Isolated zooplankton from each tank were then divided into two sub-samples using a plankton splitter and transferred to the two other tanks in the metacommunity. Thus, dispersal events were density-independent, symmetric and reciprocal among tanks within a metacommunity. For the 0% dispersal treatments, tanks were mixed, but no samples were taken.

All tanks experienced semi-continuous replacements of water every 2–3 days (the day before dispersal events). Tanks were mixed and 30 l was removed using a 10 l bucket. Water was then replaced with well water with dissolved phosphorus and nitrogen added at target levels (as above). The 30 l of water removed for replacements was used as a plankton sample by filtering out zooplankton using a sieve and preserving the isolate in 90% ethanol. Samples were later enumerated using a stereomicroscope. Zooplankton responses were quantified twice weekly, beginning on day 14 and ending on day 74, the last day of the experiment. To determine clonal composition of *Daphnia* populations, microsatellite analyses were performed on a sub-sample of 40 to 48 haphazardly chosen individuals taken from the ethanol-preserved samples (see electronic supplementary material, Methods). Clones were identified as multi-locus genotypes based on unique combinations of alleles for four microsatellite loci. Frequencies (relative abundances) of clones were estimated for each sub-sample. Clonal dynamics were quantified once weekly beginning on day 18 and ending on day 74 of the experiment. In some instances, *Daphnia* population densities were too low to obtain an adequate number of individuals for microsatellite analyses. To ensure that estimates of clonal diversity were based on comparable sampling efforts, we excluded samples with less than 40 individuals from our analyses of diversity.

### 2.2. Statistical analysis

All analyses were performed using R v. 3.6 [34]. R code is available in the electronic supplementary material, Methods. To facilitate comparisons between the 0% dispersal treatment and dispersal-present treatments and to ensure that comparisons were made among systems of equal volume, we used a target-neighbour approach in which responses from a single tank from each metacommunity were used for comparisons among treatments. We arbitrarily chose the left-most tank in each metacommunity when facing south as the target tank. Metacommunities were randomly positioned in the field, reducing bias introduced by our method of tank choice. Species diversity for each sample date was quantified using richness (based on presence/absence of taxa). As changes in diversity may have also been generated by changes in species evenness, we used two common measures of diversity that simultaneously account for species relative abundances and richness: the inverse Simpson index and the Shannon index. To analyse responses within *Daphnia* populations, clone frequencies were first multiplied by total *Daphnia* density to obtain clone abundances for each tank and sample date. Clonal diversity was calculated for each sample date using the same metrics used for species diversity. Diversity measures were averaged over time for analysis of treatment effects. To quantify temporal variability of zooplankton populations and clone abundances, the coefficient of variation (CV) of densities over time was used as an inverse measure of temporal stability. Theory predicts that dispersal can influence both steady-state dynamics and transitory dynamics of populations experiencing exponential growth [12,14]. Consequently, we analysed temporal stability using all sample dates which included an initial population growth phase in the first half of the experiment. One zooplankton species, *Diaphanosoma*, and several clones were extremely rare in some treatments and replicates. Time-series composed of zeroes and a single non-zero value can lead to biased and misleading results when using the CV. Consequently, we limited analyses to cases where the species or clone had two or more non-zero values in each replicate.

For analyses, dispersal was expressed as average proportion of tank volume per day (0, 0.01, 0.03 and 0.06 per day). Effects of dispersal on responses were analysed using linear regression. In several cases, residual plots indicated deviations from linearity. For these, we fitted an asymptotic relationship using ordinary least-squares nonlinear regression and the SSasymp self-start regression function in R. This function models the response variable *y* (i.e. measures of diversity and temporal variability) as a function of the predictor *x* (i.e. dispersal rate) as: *y* = Asym + (*y*_o_ − Asym) × exp( − exp(*z*) × *x*), where Asym is the asymptotic value of *y* at large values of *x, y*_o_ is the value of *y* when *x* equals zero, and *z* is the natural log of the rate constant (governing the rate at which the relationship approaches Asym with increasing *x*). We considered models that produced significant (*p* < 0.05) estimates of Asym and/or *y*_o_ as statistically significant. Nonlinear model results are only shown for cases where the nonlinear regression improved plots of residuals (reducing trends). For cases where significant asymptotic relationships were detected, *post hoc* pairwise comparisons among dispersal levels were performed using Tukey's HSD test to further elucidate declining effects of dispersal on the response. Model assumptions were assessed using plots of residuals, normal q–q plots and Shapiro's test of normality. Assumptions of the nonlinear models were assessed using the nlstools package. Several responses required log transformation to meet assumptions of normality of errors. All tests met assumptions of normality, but many intraspecific responses displayed potential violations of homogeneity of variances which could not be remedied with transformation. Thus, *p*-values should be viewed with caution for these results.

## 3. Results and discussion

Two aspects of habitat fragmentation are frequently cited as primary drivers of diversity loss: increased habitat isolation and reduced local habitat size. Separating the effects of these two mechanisms in nature is a challenge, and controversy persists regarding their relative importance [6,8,35]. By maintaining constant local patch size and directly manipulating dispersal rates, we were able to remove the influence of changing patch size and elucidate the dispersal effects of isolation on population and sub-population responses. We show clearly that increasing isolation and reduced dispersal rates can have significant impacts on biodiversity and stability measured at both inter- and intraspecific levels.

**Figure 1.**
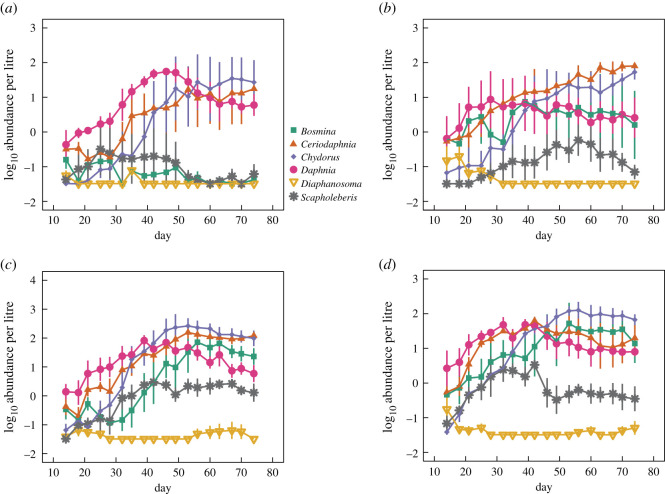
Zooplankton population dynamics in the four dispersal rate treatments: (*a*) 0.00 per day, (*b*) 0.01 per day, (*c*) 0.03 per day and (*d*) 0.06 per day. Shown are mean log_10_ transformed abundances over time of the six species used in the experiment (±s.e.). A constant equal to the sampling detection limit was added to zero values for display purposes.

**Figure 2.**
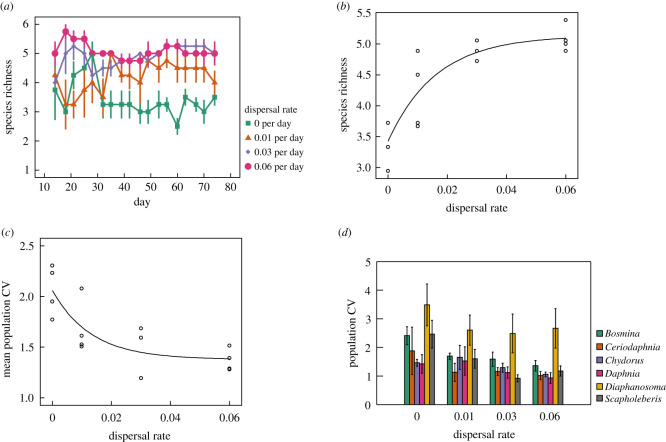
Effects of dispersal rate on interspecific diversity and temporal variability (an inverse measure of stability). (*a*) Changes in species richness over time for the four dispersal rate treatments (means ± s.e.). (*b*) The effect of dispersal rate on time-averaged species richness (shown is the nonlinear regression fit). (*c*) The effect of dispersal rate on population-level temporal variability (measured as the CV) averaged across all species (shown is the nonlinear regression fit). (*d*) The effect of dispersal rate on population-level temporal variability for each species (means ± s.e.).

Effects of isolation were strongest when examining interspecific responses. Figure 1 displays population dynamics across dispersal treatments. In the absence of dispersal, communities were dominated by three taxa: *Daphnia*, *Ceriodaphnia* and *Chydorus* (figure 1*a*; electronic supplementary material, figure S1). Reduced isolation and increasing dispersal rate appeared to enhance diversity and evenness among zooplankton taxa by increasing the abundances of *Scapholeberis* and *Bosmina*, sustaining their densities over the course of the experiment (figure 1*b*–*d*; electronic supplementary material, figure S1). Consistent with theoretical predictions, increasing dispersal rate had a positive effect on species richness relative to completely isolated communities, especially in the latter half of the experiment (figure 2*a*). When analysing time-averaged species richness, effects of increasing dispersal were significant using nonlinear regression (figure 2*b*; *p* < 0.0001, residual standard error = 0.361). The asymptotic relationship indicated that positive effects on species diversity weakened with increasing levels of dispersal. *Post hoc* pairwise comparisons among dispersal levels showed that mean species richness was lower in fully isolated communities compared to the three treatments with dispersal present (all *p* < 0.058, Tukey's HSD). Species richness in the 0.01 dispersal treatment was significantly lower than the 0.06 treatment (*p* = 0.026, Tukey's HSD), but no difference was detected when comparing the 0.01 and 0.03 dispersal treatments (*p* = 0.091, Tukey's HSD) or the 0.03 and 0.06 dispersal treatments (*p* = 0.882, Tukey's HSD). Similar results were obtained when measuring species diversity as time-averaged Shannon diversity (electronic supplementary material, figure S2; *p* < 0.0001, residual standard error = 0.111, nonlinear regression) and inverse Simpson diversity (electronic supplementary material, figure S3; *p* < 0.0001, residual standard error = 0.224, nonlinear regression). A caveat to consider when interpreting our results is that a small amount of phytoplankton and bacteria was likely transferred with zooplankton during dispersal events. Thus, zooplankton responses may have been influenced by dispersal effects on their resources. As we did not sample phytoplankton and bacterioplankton communities in our study, we cannot directly address this possibility.

Reduced isolation also enhanced temporal stability measured at the population level. When averaging across taxa, temporal variability declined with increasing dispersal rate (figure 2*c*; *p* < 0.0001, residual standard error = 0.243, nonlinear regression). Thus, reduced isolation (i.e. increasing dispersal rate) enhanced mean population-level temporal stability by reducing the variability of abundances through time. Much like species diversity responses, results indicated an asymptotic relationship with diminishing effects of increasing dispersal on mean temporal variability. When performing *post hoc* pairwise comparisons among individual treatment levels, mean temporal variability in the 0.00 treatment did not differ from the 0.01 dispersal treatment (*p* = 0.19, Tukey's HSD), but was significantly lower than the 0.03 and 0.06 treatments (*p* < 0.01, Tukey's HSD). No significant differences in temporal variability were detected among the 0.01, 0.03 and 0.06 dispersal treatments (all *p* > 0.34, Tukey's HSD). Figure 2*d* displays measures of temporal variability for individual species in response to the dispersal treatments. We predicted that all taxa would show declines in temporal variability with increasing dispersal rate. While several taxa exhibited declining trends in temporal variability with increasing dispersal (figure 2*d*; electronic supplementary material, figure S4), effects were strongest for species whose abundances were most benefited by dispersal: *Scapholeberis* (figure 2*d*; electronic supplementary material, figure S4; *p* < 0.0001, residual standard error = 0.517, nonlinear regression) and *Bosmina* (figure 2*d*; electronic supplementary material, figure S4; *p* < 0.0001, residual standard error = 0.373, nonlinear regression), in addition to *Chydorus* (figure 2*d*; electronic supplementary material, figure S4; *p* = 0.025, *r*^2^ = 0.26, linear regression). Effects of dispersal rate on temporal variability were not significant for *Daphnia* (electronic supplementary material, figure S4; *p* = 0.11, linear regression), *Ceriodaphnia* (electronic supplementary material, figure S4; *p* = 0.21, linear regression) or *Diaphanosoma* (electronic supplementary material, figure S4; *p* = 0.54, linear regression).

These results complement a growing body of empirical research showing that decreasing connectivity and reduced dispersal rates can negatively impact local species diversity [6,13,36–40] and population-level temporal stability [41,42]. As the zooplankton species used in our study are found in the same ecological guild of filter feeders, interspecific competition likely played a major role in structuring local communities and dynamics. Hence, our findings are consistent with models that show that dispersal can promote local species diversity by weakening interspecific competition and preventing local competitive exclusion [10,15,16]. These same models predict that positive effects of dispersal on species diversity may diminish and even reverse with increasing rates of movement, reducing local diversity at extremely high levels of immigration. While we found no evidence of a decline in species diversity at our highest level of dispersal, we did detect asymptotic relationships, indicating declining effects of dispersal and reduced isolation. A potential concern is that the dispersal rates used in our experiment may have been low when compared to natural rates of zooplankton movement. Few studies have attempted to quantify zooplankton dispersal rates in nature. However, in a study of a pond metacommunity in which systems were directly connected by streams, the maximum observed proportional emigration rate was 0.009 per day [43]. Given that zooplankton are passive dispersers, dispersal rates are likely much lower among insular ponds not directly linked by waterways. Thus, it is highly plausible that the two highest dispersal rates used in our experiment greatly exceeded rates of movement in many natural pond metacommunities.

**Figure 3.**
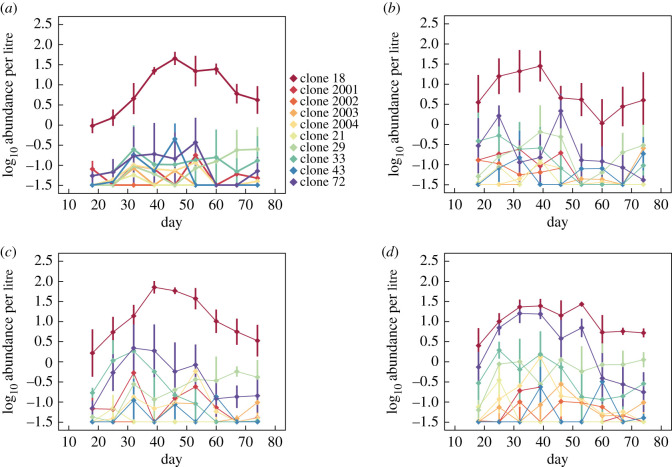
*Daphnia* clonal dynamics in the four dispersal rate treatments: (*a*) 0.00 per day, (*b*) 0.01 per day, (*c*) 0.03 per day and (*d*) 0.06 per day. Shown are mean log_10_ transformed abundances over time for the 10 clones detected in the experiment (±s.e.). A constant equal to the sampling detection limit was added to zero values for display purposes.

**Figure 4.**
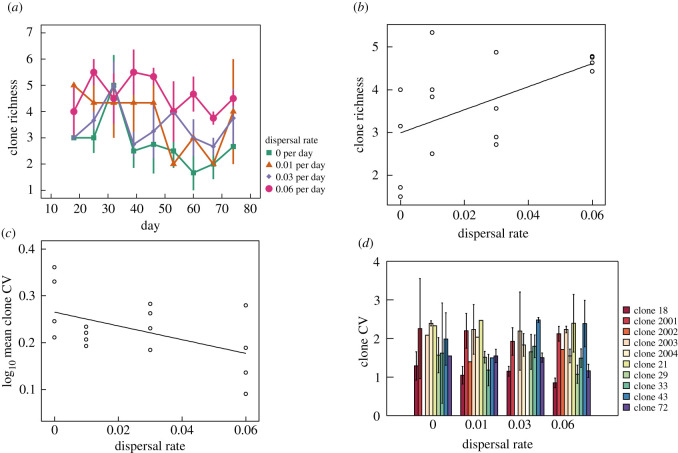
Effects of dispersal rate on intraspecific diversity and temporal variability (an inverse measure of stability). (*a*) Changes in *Daphnia* clone richness over time for the four dispersal rate treatments (means ± s.e.). (*b*) The effect of dispersal rate on time-averaged *Daphnia* clone richness (shown is the linear regression fit). (*c*) The effect of dispersal rate on log_10_ transformed temporal variability (measured as the CV) of *Daphnia* clone abundances averaged across all clones (shown is the linear regression fit). (*d*) The effect of dispersal rate on temporal variability of clone abundances for each *Daphnia* clone detected in the experiment (means ± s.e.).

Prior studies have shown that habitat fragmentation can also reduce the genetic diversity of local populations [25,26]. However, few have explored how habitat isolation and dispersal impact intraspecific diversity and genotype dynamics at shorter, ecological time scales [18,19,27]. For many zooplankton species such as *Daphnia pulex*, intra-annual dynamics are dominated by asexual reproduction. Hence, ecological processes that influence interspecific diversity and stability may also simultaneously drive intraspecific clonal dynamics. In our study, intraspecific diversity responses paralleled interspecific responses, but patterns were overall weaker and linear in form. Figure 3 displays clonal dynamics across dispersal treatments. Of the 10 clones used in our experiment, *Daphnia* populations were numerically dominated by a single genotype, clone 18, in the absence of dispersal (figure 3*a*; electronic supplementary material, figure S5). Decreasing isolation (i.e. increasing dispersal rate) appeared to enhance clonal diversity and evenness by increasing the abundance of several rarer clones—most notably clones 29, 33 and 72 (figure 3*b*–*d*; electronic supplementary material, figure S5). As predicted, dispersal rate enhanced clonal diversity, measured as richness, over the course of the experiment (figure 4*a*). When analysing clonal richness averaged over time, a significant positive effect of dispersal rate was detected (figure 4*b*; *p* = 0.015, *r*^2^ = 0.31, linear regression). Similar results were obtained when measuring clonal diversity using the Shannon index (electronic supplementary material, figure S6; *p* = 0.027, *r*^2^ = 0.30, linear regression) and the inverse Simpson index (electronic supplementary material, figure S7; *p* = 0.034, *r*^2^ = 0.23, linear regression).

Much like interspecific responses, reduced isolation and increasing dispersal rate stabilized the dynamics of *Daphnia* genotypes within patches when averaging across clones. A significant negative relationship was detected between dispersal rate and mean temporal variability (figure 4*c*; *p* = 0.05, *r*^2^ = 0.25, linear regression). Figure 4*d* displays measures of temporal variability for individual clones in response to the dispersal treatments. While we predicted that increasing dispersal would enhance stability of all clones, we found that patterns in figure 4*c* were driven by a minority of clones, most notably clones 72 and 2004 (figure 4*d*). When analysing individual clone CVs, only clone 72 (figure 4*d*; electronic supplementary material, figure S8; *p* = 0.03, *r*^2^ = 0.38, linear regression) and clone 2004 (figure 4*d*; electronic supplementary material, figure S8; *p* < 0.01, *r*^2^ = 0.77, linear regression) exhibited significant negative relationships between temporal variability and dispersal. Several clones were rare and absent from multiple replicates or, in some cases, entire treatment levels, limiting statistical power.

While fragmentation is widely recognized as a significant threat to biodiversity, research on this topic has historically had a terrestrial focus. There is growing recognition of the importance of such impacts in shallow freshwater ecosystems such as wetlands and ponds [37,44,45]. These systems are known to be important sources of vertebrate and invertebrate biodiversity [46]; hence, elucidating the drivers of biodiversity loss in shallow waterbodies is vital from a conservation perspective. For passive dispersers such as zooplankton, increasing habitat isolation could reduce zooplankton dispersal rates among patches by reducing immigration/emigration rates of animal vectors (e.g. by increasing travel time among habitats). Our study was clearly a simplified representation of such processes, using direct manipulations of dispersal that were reciprocal and density-independent in form. Effects of habitat isolation in nature may be more complex and involve numerous underlying mechanisms, including changes in patterns of landscape structure, patch connectivity and dispersal network structure [13,47]. Moreover, the quality of habitats surrounding local patches (the habitat matrix) may also influence dispersal capacity via effects on abundance, mortality, composition and movement rates of vectors [48]. Our study approximated such effects in an artificial way through direct manipulations of dispersal rates. Furthermore, by using reciprocal dispersal among patches, we were able to test the effects of dispersal rate independent of changes in patterns of connectivity. How alterations in connectivity and dispersal network structure impact local biodiversity and stability is an important question and an aspect of habitat isolation that warrants further experimental exploration in our study system. Despite these limitations, our work showed that increasing habitat isolation, in the form of reduced dispersal rates, can be an important driver of biodiversity loss and instability in pond planktonic systems, effecting declines in diversity and temporal stability measured at both inter- and intraspecific levels. Further research is needed to determine how our findings relate to long-term, inter-annual patterns of diversity. Nonetheless, the processes uncovered in our study clearly have the potential to contribute to the long-term maintenance of zooplankton biodiversity and highlight the destructive role of isolation on the persistence and adaptive capacity of populations.

## Data accessibility

Data are available in Dryad: https://doi.org/10.5061/dryad.0vt4b8h18. The data are provided in the electronic supplementary material [49].
